# Digital dermatitis in cattle is associated with an excessive innate immune response triggered by the keratinocytes

**DOI:** 10.1186/1746-6148-9-193

**Published:** 2013-10-03

**Authors:** Walid Refaai, Richard Ducatelle, Peter Geldhof, Belgacem Mihi, Mahasen El-shair, Geert Opsomer

**Affiliations:** 1 Department of Reproduction, Obstetrics, and Herd Health, Faculty of Veterinary Medicine, Ghent University, Salisburylaan 133, 9820, Merelbeke, Belgium; 2 Department of Pathology, Bacteriology and Avian Diseases, Faculty of Veterinary Medicine, Ghent University, Salisburylaan 133, 9820, Merelbeke, Belgium; 3 Department of Virology, Parasitology and Immunology, Faculty of Veterinary Medicine, Ghent University, Salisburylaan 133, 9820, Merelbeke, Belgium; 4 Department of Surgery, Anesthesiology and Radiology, Faculty of Veterinary Medicine, Zagazig University, Zagazig, Egypt

**Keywords:** Digital dermatitis, Pathogenesis, Keratinocytes, Cytokines, Cattle

## Abstract

**Background:**

Digital Dermatitis (DD) is a common disease of dairy cows, the pathogenesis of which is still not clear. This study examined some host responses associated with the typical lesions, in an attempt to further elucidate the pathogenesis of the disease. Twenty four samples representing the 5 different clinical stages of DD (M0-M4) were collected from slaughtered cattle for histopathological and immunological analyses.

**Results:**

Significant increases in total epidermal thickness were found in M2, M3, and M4 when compared with M0 and M1. M3 samples, when compared with M0 and M1, were characterized by a significant increase in the thickness of the keratin layer. Counts of both eosinophils and neutrophils were at a maximum in the M2 stage and decreased in the M3 and M4 stage. A significant increase in IL8 expression was observed in the M2-M3 stages of the disease and immunohistochemical staining showed the source as keratinocytes, suggesting an important role for keratinocyte-derived IL8 in the pathogenesis of DD.

**Conclusion:**

Results of the present study point to a strong stimulation of the innate immune response at the level of the keratinocytes throughout most of the clinical stages, and a delayed response of the adaptive immune reaction.

## Background

Digital Dermatitis (DD) is currently one of the main problems of the underfoot in cattle, both because of its high prevalence as well as because of it is painful and leads to significant welfare problems and economic losses for the livestock industry [1]. The disease was first reported in Italy by Cheli and Mortellaro [2], and has since then been recognized as an universal cause of lameness in cattle in many countries all over the world [3].

The disease is characterized by a superficial dermatitis of the distal part of the foot. Lesions present as painful, circular or oval erosions and may become granular, strawberry-like or rapidly progress to filiform warts with matted hair [4]. Finally, healing is characterized by the presence of a dry, painless crust tightly adherent to the underlying healthy skin [4,5]. Lesions are mostly located at the skin bordering the interdigital space on the palmar/plantar aspect of the foot, especially the hind foot, at the skin–horn junction of the heel bulbs or along the coronary band [4]. The skin bordering the dorsal interdigital cleft of the foot may also be involved [6,7]. In some papers, typical DD lesions are described also in the area of the dewclaws [7]. Döpfer et al. [8] classified clinical DD lesions into five different stages (M0- M4), mainly based on visual inspection of the lesions. M0 represents the normal digital skin without DD lesions; M1, is characterized by an early small pink area of < 2 cm in diameter; M2, is characterized by an acute classical ulcerative lesion >2 cm in diameter; M3, represents the healing stage where the lesion typically is covered by a firm scab; and M4 represents the late chronic stage where the lesion is characterized by dyskeratosis or a proliferative overgrowth or both. Few papers describe the histopathology of DD lesions. Representative lesions are reported to be characterized by a thickening of the epidermis with superficial necrosis and hyperkeratosis, elongation of the rete ridges, and infiltration of neutrophils, macrophages, lymphocytes, and eosinophils in both the epidermal and dermal layers [7,9]. Until now however, these histopathologic features have not been matched with the clinical staging as described by Döpfer et al. [8].

Digital dermatitis is considered a multifactorial disease with a strong bacterial component [10]. Best candidate etiological bacterial agents of DD are *Treponema spp*[11,12]. Epidemiologic studies indicate the presence of an excessive amount of mud or a generally moist environment as a paramount risk factor [13], which was recently confirmed in a successful experimental infection model [14]. Dairy cows appear to be at a significantly higher risk in comparison to beef cows, the incidence of the lesions being highest near peak lactation [15,16]. Although the difference in incidence between dairy versus beef cows may be caused by differences in housing, recent studies suggest differences in susceptibility to be partly based on genetic backgrounds [17-19].

Although the disease is known for almost 40 years and a lot of papers have been dedicated to examining this disease, the exact pathogenesis is still not completely clear [10]. In this context, currently a lot of attention goes towards the reaction of the host in the establishment of the typical lesions, and in this respect the genetic makeup of the afflicted animal has recently been suggested to be of considerable importance [20].

The main aim of the present study was to histopathologically and immunologically examine representative DD lesions belonging to the different clinical stages (M0-M4), in order to get a better insight into the host response associated with the typical lesions, in an attempt to further elucidate the pathogenesis of the disease.

## Methods

### Tissue collection

Samples (n=24) representing the normal (=M0) and four different clinical stages of DD (M1-M4; as Döpfer et al. [8]) were collected from feet of slaughtered cattle at the slaughter house. The history of the slaughtered cows and collected DD lesions was unknown. Collected samples were taken as soon as possible after slaughter (variation: 1 to 5 hours following slaughter) either from the dorsal skin (n=10) or from the palmar/plantar skin (n=14) bordering the interdigital space. The number of M0, M1, M2, M3, and M4 samples examined was 4, 5, 5, 4, and 6 respectively. Classic full-thickness skin biopsies (2 cm in size) from the DD lesions were taken for both histopathological (10% neutral buffered formalin) and immunological analyses (immediately preserved in liquid nitrogen and subsequently stored at -80°C until RNA was extracted).

### Histopathology

**Figure 1:**
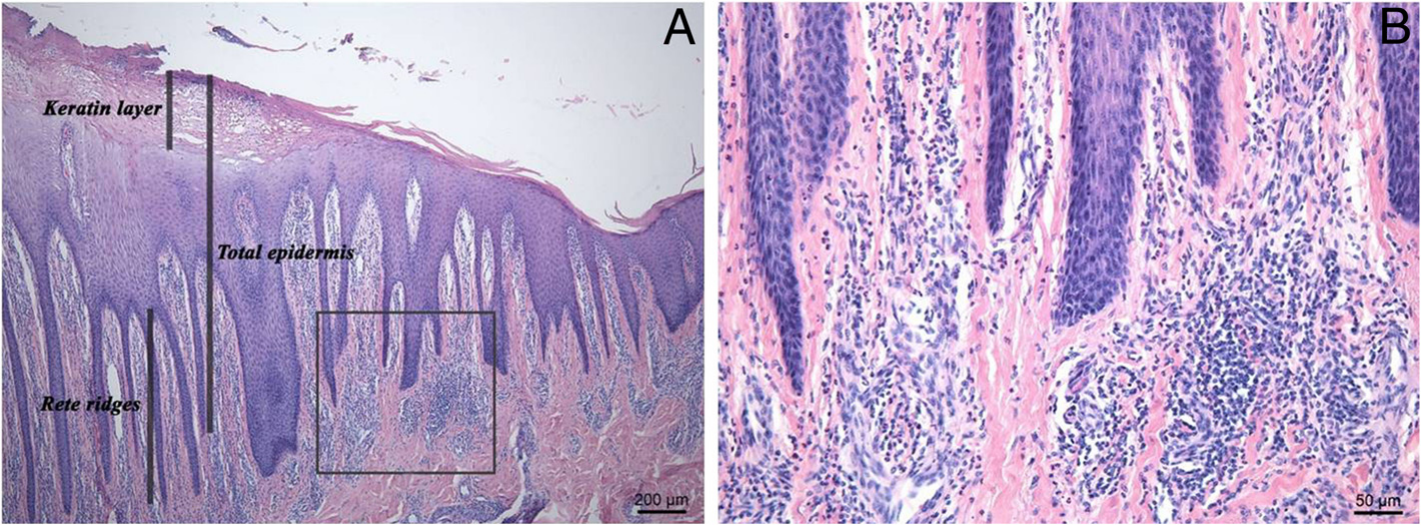
**HE staining of the digital skin affected by a digital dermatitis lesion (M4).** Thickening of the epidermal layer and elongation of rete ridges **(A)** and infiltration of eosinophils, neutrophils, and lymphocytes in the dermis at the junction with the epidermis **(B)**.

Tissue specimens were embedded in paraffin wax, and sections of 5 μm thickness were cut and stained with hematoxylin and eosin (HE) and examined by light microscopy. Total epidermal thickness, the keratin layer thickness, and the length of the rete-ridges were measured at 25× or 50× magnification at 10 different randomly selected parts of the lesion (Figure 1). Neutrophils and eosinophils, identified by their morphology under the light microscope, were counted in twenty randomly selected fields at 400× magnification. Cells were counted as proportion of the total cell number in the dermis just underneath and along the basal layer of the epidermis.

### Immunohistochemistry

Serial paraffin sections were cut at 5 μm thickness and mounted onto APES-coated glass slides. After de-paraffination and rehydration, antigen retrieval was achieved using sodium citrate. Hydrogen peroxide was used to block the endogenous peroxidase activity for 5 minutes. Sections were incubated 30 min at room temperature with anti-hCD_3_ (rabbit polyclonal, Dako, Glostrup, Denmark) or anti-hCD_20_ (rabbit polyclonal, Thermo Scientific, Fremont, USA). For detection of IL8, sections were incubated with anti-sheepIL8 (mouse monoclonal, Abcam, Cambridge, UK) overnight at 4°C. Thereafter, slides were incubated with Envision+system-HRP (Dako) for 30 min. Finally, sections were covered with DAB (Dako) for 5 minutes. Counterstaining with hematoxylin was performed and sections were dehydrated and mounted for microscopic examination. Negative control staining was performed by the same procedure except that the primary antibodies were replaced by buffer. For T and B lymphocyte counting, ten random fields at 400x total magnification were taken from each slide stained for CD_3_ and CD_20_ respectively. Cells were counted in the dermal area adjacent to the epidermis.

### RNA extraction, cDNA synthesis and quantitative Real-time PCR

RNA extraction, cDNA synthesis, and quantitative real-time PCR (qRT-PCR) were performed as described in Mihi et al. [21]. Briefly, Trizol (Invitrogen, Gent, Belgium) was used to extract the total RNA from the homogenized frozen tissue samples. Total RNA was purified using the RNeasy Mini kit (Qiagen, Venlo, The Netherlands) according to the manufacturer’s instructions. Thereafter, genomic DNA (gDNA) was removed using RNase-free DNase set (Qiagen, Venlo, The Netherlands). Genomic DNA contamination was checked using intron-spanning primers for Glyceraldehyde-3-phosphate dehydrogenase (GAPDH). Finally, one μg of total RNA was converted to cDNA using the iScript cDNA synthesis kit (Bio-Rad, Nazareth, Belgium), following the manufacturer’s instructions.

Quantitative Real-time PCR (qRT-PCR) was performed to detect the gene expression for the following genes: Chemokine receptor-3 (CCR3); Interleukin-1β (IL-1β); Interleukin-4 (IL4); Interleukin-5 (IL5); Interleukin-6 (IL6); Interleukin-8 (IL8); Interleukin-10 (IL10); Interleukin-13 (IL13); Interleukin-17 (IL17); Interferon γ (IFN-γ); Transforming growth factor beta (TGF-β); Tumor necrosis factor alpha (TNF-α); and Ribosomal protein large PO (RPLPO). The primers used in the PCR work were designed using the Primer3 software (http://frodo.wi.mit.edu/primer3/) and are shown in Table 1.

**Table 1:** Genes used in qRT-PCR work, referring to the GeneBank accession number and primer sequences

**Gene**	**Primer sequence**	**Accession number**	**Ta (°C)**
CCR3	F: TGTGTCAACCCCGTGATCTA	NM_001194960.1	60
	R: AGAGTTCCTGCTCCCCTGTT		
IL1β	F: AAGGCTCTCCACCTCCTCTC	NM_174093.1	60
	R: TTTGGGGTCTACTTCCTCCA		
IL4	F: GCGGACTTGACAGGAATCTC	NM_173921.2	64
	R: TCAGCGTACTTGTGCTCGTC		
IL5	F: TGGTGGCAGAGACCTTGACA	NM_173922.1	60
	R: TTCCCATCACCTATCAGCAGAGT		
IL6	F: TCCTTGCTGCTTTCACACTC	NM_173923.2	60
	R: CACCCCAGGCAGACTACTTC		
IL8	F: GTTGCTCTCTTGGCAGCTTT	NM_173925.2	60
	R: GGTGGAAAGGTGTGGAATGT		
IL10	F: TGTATCCACTTGCCAACCAG	NM_174088.1	60
	R: CAGCAGAGACTGGGTCAACA		
IL13	F: GGTGGCCTCACCTCCCCAAG	NM_174089.1	60
	R: GATGACACTGCAGTTGGAGATGCTG		
IL17	F: GGACTCTCCACCGCAATGAG	NM_001008412.1	60
	R: TGGCCTCCCAGATCACAGA		
IFNϒ	F: TTCTTGAATGGCAGCTCTGA	NM_174086.1	60
	R: TTCTCTTCGGCTTTCTGAGG		
TGFβ	F: CTGCTGTGTTCGTCAGCTCT	NM_001166068.1	60
	R: TCCAGGCTCCAGATGTAAGG		
TNFA	F: GCCCTCTGGTTCAGACACTC	NM_173966.2	60
	R: AGATGAGGTAAAGCCCGTCA		
GAPDH	F: GGGTCATCATCTCTGCACCT	NM_001034034.1	60
	R: GGTCATAAGTCCCTCCACGA		
RPLP0	F: CTTCATTGTGGGAGCAGACA	NM_001012682.1	60
	R: GGCAACAGTTTCTCCAGAGC		

F: forward primer.

R: reverse primer.

Ta: annealing temperature.

Amplification reaction was carried out using the StepOnePlus Real-Time PCR System (Applied Biosystems, Ghent, Belgium) using the SYBR Green Master Mix (Applied Biosystems, Ghent, Belgium). Relative changes in the gene expression of the different tested genes for both the normal and diseased groups were carried out by using the 2^-ΔΔ^*C*_T_ method [22]. Ct values were transformed into relative quantity (Q value) using the delta Ct method and Q values of the tested genes were normalized against that of a housekeeping gene (RPLPO). Normalized Q values were used in the statistical analysis.

### Statistical analysis

Data were statistically analyzed using GraphPad Prism software. The nonparametric Mann Whitney *U* test was used to determine variations in the gene expressions between diseased groups versus the normal one. For the histopathological measurements and cell counts, the effect of the clinical stage was investigated using the One Way Anova test. Pair-wise comparisons were made using the Tukey's multiple comparisons test. Results were formulated as (mean ± SEM). Spearman's rank correlation was used to investigate the relationships between the gene expressions, histopathological measures, and cell counts. In all cases, an associated probability (P-value) of < 0.05 was considered significant.

## Results

### Measurements of total epidermal and keratin layer thickness and rete ridges length

**Figure 2:**
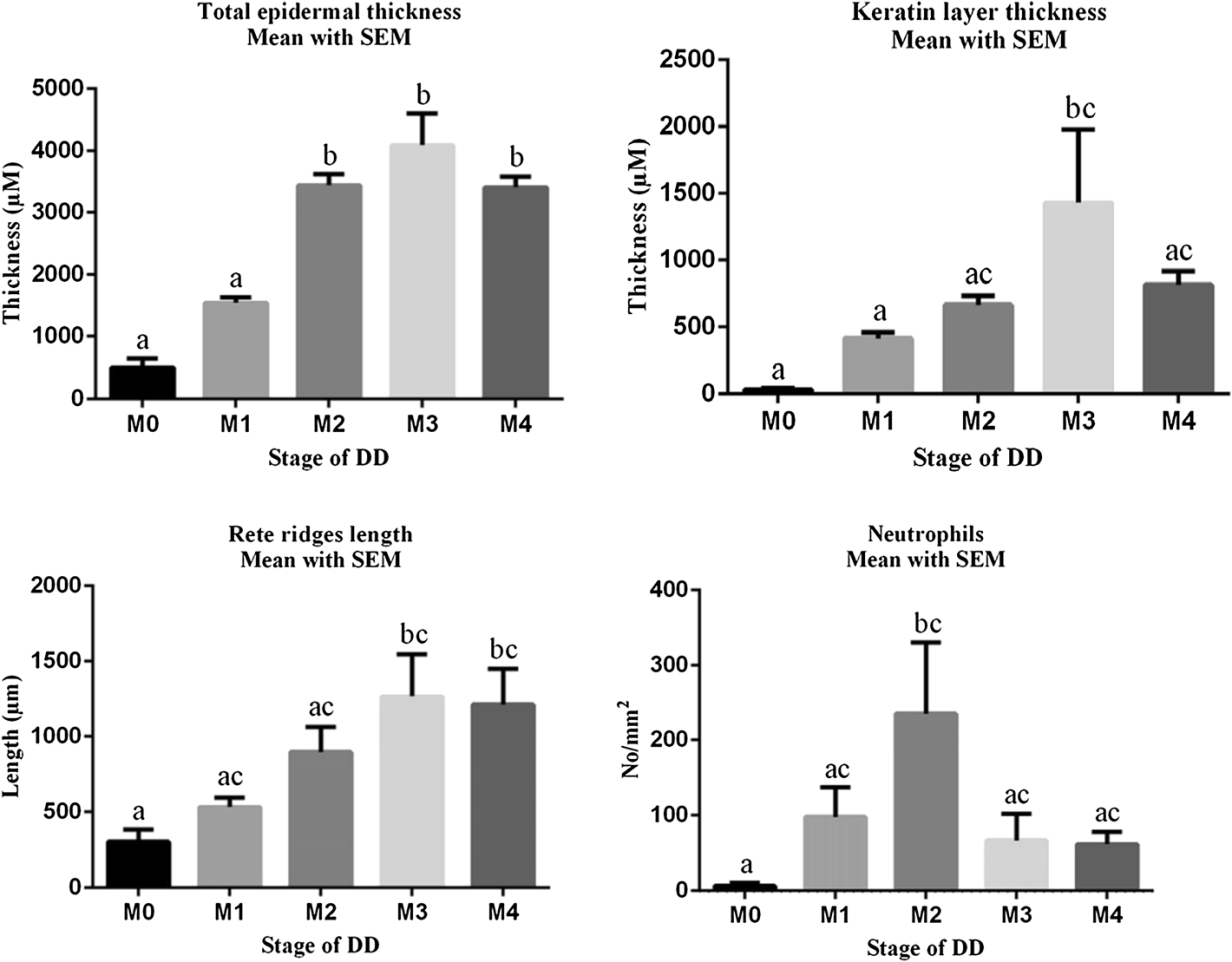
**One Way Anova test used to statistically represent the measurements of the total epidermal and keratin layer thickness, length of rete ridges, and the number of neutrophils infiltrated in the dermis adjacent to the epidermis in the normal compared to the diseased groups.** Differences between groups with different letters are significant (P value < 0.05).

Results show that the thickness of the total epidermis and keratin layer, and the length of the rete ridges increased gradually from M0 up to M3 and subsequently tended to decrease again in the M4 stage (Figure 2). Small focal ulcerative areas were detected both in M2 and M3 stages.

Significant increases in total epidermal thickness were found in M2, M3, and M4 when compared with M0 and M1. M3 samples, when compared with M0 and M1, were characterized by a significant increase in the thickness of the keratin layer. The length of the rete ridges in M3 and M4 was significantly increased in comparison with M0.

### Counting immune cells (Eosinophils, Neutrophils, Lymphocytes)

**Figure 3:**
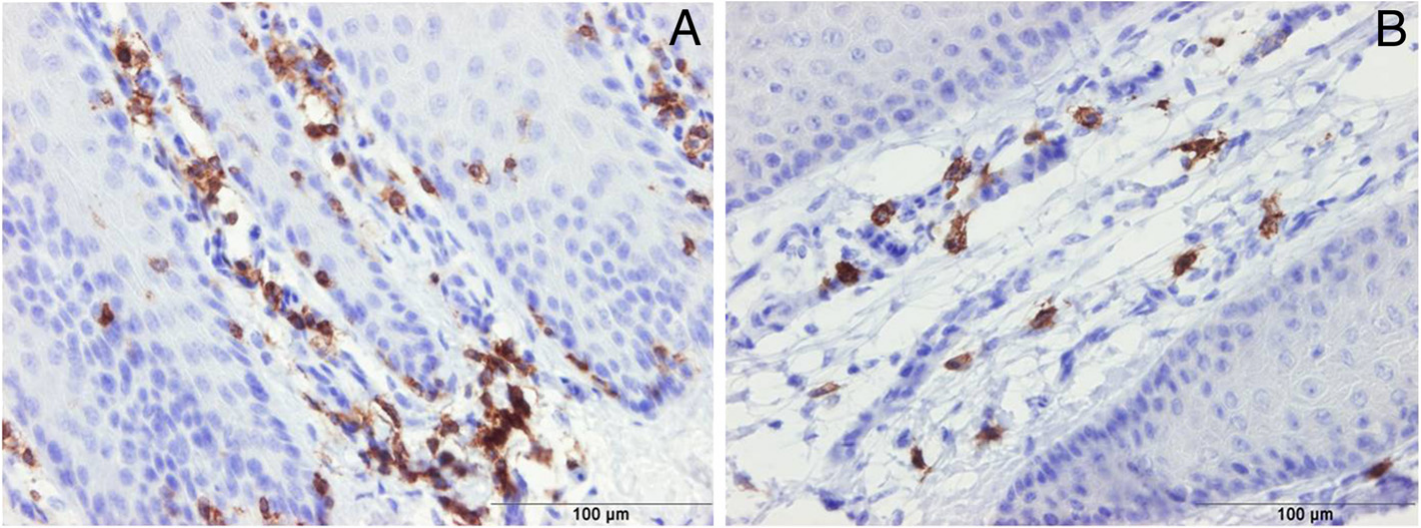
**Immunohistochemical staining of digital dermatitis infected skin for detection of CD**_**3 **_**(+ve T-lymphocytes ) (A, M0) and CD**_**20 **_**(+ve B-lymphocytes) (B, M4**)**.** T and B lymphocytes were observed mainly in the dermis at the junction with the epidermis.

Hematoxylin-eosin and immunohistochemically stained sections showed a high number of infiltrating immune cells (eosinophils, neutrophils, and lymphocytes) in the reticular dermis (Figures 3 and 2). Numbers of both eosinophils and neutrophils reached a maximum in the M2 stage and subsequently decreased in the M3 and M4 stage. The number of neutrophils measured in the M2 stage was significantly higher than in the M0 stage. High numbers of T lymphocytes were counted in all DD stages including M0 without any significant difference between the different stages. The number of B-lymphocytes was highest in the M4 stage although differences found between the different M stages were not significant.

### Quantitative Real-time PCR

The gene expression of 10 cytokines, one chemokine, and one chemokine receptor was measured by Quantitative Real-time PCR (qRT-PCR) both in the normal as well as in the diseased groups and the results are shown in Table 2. Cytokines included in the analysis were classified into pro-inflammatory cytokines (IL1B, IL6, TNF-α), Th1 type cytokines (IFN-γ), Th2 type cytokines (IL4, IL5, IL13), T regulatory cytokines (IL10, TGF-β) and Th17 type cytokine (IL17). Besides, a chemokine receptor-3 (CCR3) and proinflammatory chemokine (IL8) were also analyzed.

**Table 2:** Transcription levels of different genes in digital dermatitis diseased groups (M1, M2, M3, M4) expressed as fold changes compared to the normal group (M0)

**Gene**	**M1(n=5)**	**M2(n=5)**	**M3(n=4)**	**M4(n=6)**
CCR3	0.36±0.10	0.56±0.18	0.36±0.02*	0.39±0.10*
IL1B	4.9±2.5	52.3±21.6	37.3±14.9	2.7±1.4
IL4	0.42±0.19	1.14±0.51	0.13±0.07	0.38±0.16
IL5	0.42±0.08	0.44±0.10	0.30±0.07*	0.40±0.06*
IL6	9.9±4.4	44.4±23.6	37.2±19.0	2.8±1.4
IL8	42.8±33.3	328.0±144.4*	190.7±112.7*	10.8±4.8*
IL10	1.60±0.45	2.9±0.7	1.8±0.5	1.02±0.17
IL13	0.68±0.10	0.74±0.15	0.72±0.23	0.50±0.14*
IL17	2.34±0.96	4.80±2.57	1.55±0.76	1.52±0.74
INFG	ND	ND	ND	ND
TGFB	0.83±0.16	0.95±0.16	0.63±0.09	0.68±0.12
TNFA	0.61±0.18	1.36±0.34	1.35±0.39	1.01±0.15

ND: not expressed, too low in the sample.

Data was expressed as (Mean ± SE) of fold changes in diseased groups compared to normal group.

*p-value < 0.05 is considered significant in comparison to the normal (M0) group.

n: number of samples used per group.

IL1B and IL6 transcription levels were observed to increase, reaching a peak at the M2 stage, without significant differences between the other disease stages. Moreover, a highly significant increase in the transcriptional level for IL8 was observed, with significant increases of 328 and 190 fold at the M2 and M3 stages, respectively. On the other hand, the expression levels for IL10, IL17, and TNF-α did not seem to be affected.

The transcriptional levels for IL4, IL5, IL13, and CCR3 were down-regulated in the diseased versus the control group. The same was true for the transcriptional level of TGF-β, while IFN-γ was not detectable by qRT-PCR.

### Immunohistochemical staining for IL8

**Figure 4:**
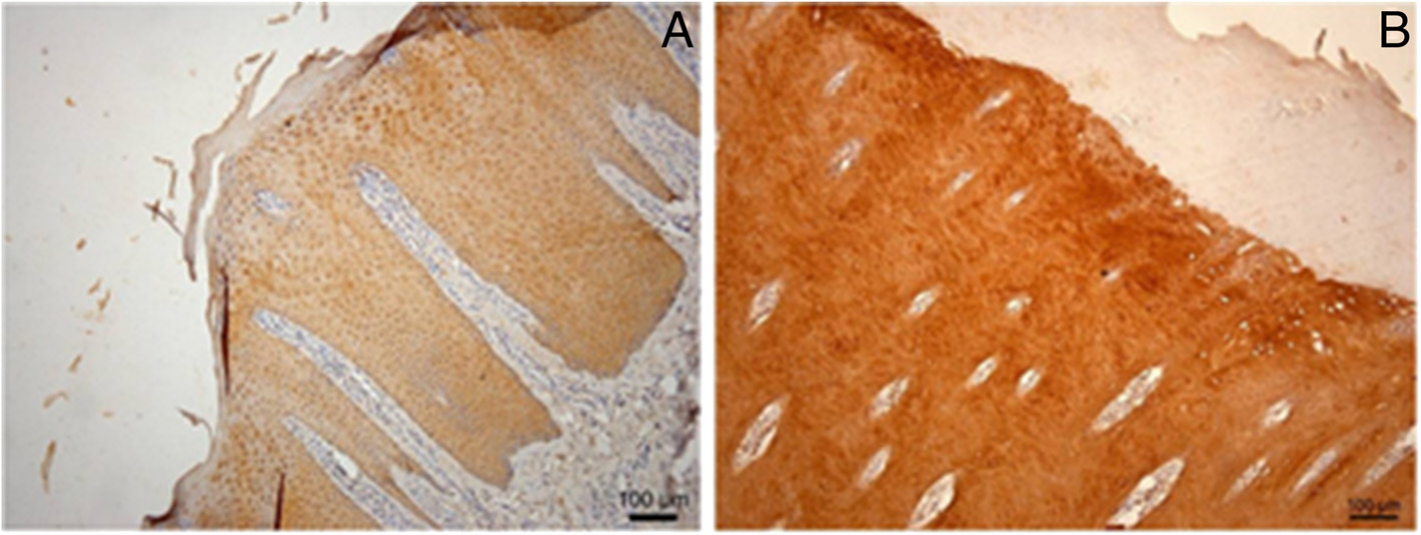
**Immunohistochemical staining of digital skin for detection of IL8.** Viable keratinocytes are the cells producing the IL8 cytokine. Both the stratum corneum and dermal papillae are not producing IL8 (**A** represents M0 and **B** represents M2).

The highly significant increase in the transcriptional level for IL8 in samples of both M2 and M3 lesions motivated us to examine which cell types were responsible for this increase. Immunohistochemical (IHC) stainings revealed that the viable keratinocytes located in the epidermis and not the cells located in the stratum corneum or dermal cells were the source of the elevated IL8 levels (Figure 4).

## Discussion

The present manuscript describes a study performed to further unravel the pathogenesis of DD in the bovine by examining the inflammatory pattern and the host response in the typical skin lesions that are associated with this painful disease.

On the HE stained sections, small ulcers in between the hypertrophied epidermis areas were detected in both M2 and M3, but not in the other stages. Coupled with the admittedly limited examination of immune cells, these data suggest that blood cells ooze out of the surface via these ulcers, either or not precipitating on the surface, giving rise to the clinical picture of a completely ulcerated lesion, or a lesion covered with a scab, as is the case in M3 according to Döpfer [8]. This suggestion would be consistent with Gomez et al. [14] as they stipulated the existence of either exposure of the stratum spinosum or ulceration of the dermis in lesions referred to as typical DD lesions. Also, previous studies have shown either a total loss of the epidermis with complete ulceration in severe cases [7], or no ulcerations but only partial loss of the epithelium in M1 and complete loss of the stratum corneum in M2 lesions [8].

Immunohistochemistry also indicated a trend in T and B cell lymphocyte distribution with the former appearing highest in normal skin and the latter in the most chronic DD stage, but this finding warrants further study. In accordance with previous studies [7-9], we found hyperplasia and hyperkeratosis of the epidermal and keratin layers and elongation of the rete ridges occurred in all diseased groups when compared with the healthy M0 samples. These findings can be attributed to a higher proliferation rate of the keratinocytes due to the acute inflammatory reaction of the skin, without a concomitant increase in differentiation and desquamation. The latter causes the epidermis in the M2 stage to be significantly increased in thickness in comparison with the M0 and M1 stages. Also the healing stage (M3) has a significantly thicker keratin layer in comparison to the M0 and M1 stages. The latter can be attributed to the formation of a firm scab during the healing process which makes the keratin layer thicker in comparison to other DD stages [8].

Infiltration of different immune cells, mainly neutrophils, eosinophils, and lymphocytes was found in the epidermal and dermal layers of the skin. This observation agrees with previous work [7-9]. The M2 stage was characterized by the highest increase in the number of infiltrating neutrophils and eosinophils. Interestingly, some skin diseases in humans, such as psoriasis, in which the immune reaction of the host is known to play a decisive role in the pathogenesis, are also characterized by a hyperplasia of the epidermis with the infiltration of different immune cells, mainly T lymphocytes and neutrophils [23].

The study showed for the first time a strong transcriptional upregulation of IL8 in the M2 and M3 stage, identifying keratinocytes as the source of this increased IL8 level.

This chemokine IL8 is known to be produced early in the inflammatory response and has a key role in the recruitment and activity of neutrophils [24,25]. IL8 is also known to have a stimulatory effect on the migration [26] and proliferation [27] of keratinocytes, which may explain the hyperplasia and thickening of the epidermal layer of the skin. The massive increase of IL8 is considered to be due not only to increased numbers of producing cells, but also to an upregulation of the expression. Previous work on psoriatic patients demonstrated that an elevated IL8 expression plays a crucial role in the pathogenesis of this human skin disease [28,29]. Currently, the role of IL8 in the pathogenesis of infectious diseases in the bovine is receiving considerable attention, since both the existence of divergent IL8 promoter heliotypes [30] as well as polymorphisms of its receptors [31,32] have recently been demonstrated. This has been shown to significantly influence the susceptibility to infectious disease and the pattern of the concomitant inflammation reaction and clearly also deserves attention in DD.

## Conclusion

Main finding of the present histopathologic study of the different clinical stages (M0 to M4) of DD in cattle, was the epidermal hyperplasia being present in all clinical stages. Concomitantly with this hyperplasia, there was a massive upregulation of the expression of IL8 in the keratinocytes which was remarkably obvious in the M2 and M3 clinical stages. The latter points to a strong stimulation of the innate immune response throughout most of the clinical stages and a delayed response of the adaptive immune reaction. Based on similarities with human skin diseases, we think that this finding may contribute to the full elucidation of the pathogenesis of this widespread disease and open the way to new treatment options.

## Competing interests

The authors declare that they have no competing interests.

## Authors’ contributions

WR, RD, PG, and GO conceived and coordinated the experiment. WR and GO collected the tissue samples used in the experiment from the slaughterhouse. WR performed the lab work under supervision and guidance of BM, RD, PG and GO. ME conceived the study, participated in its design and helped to draft the manuscript. Results were statistically analyzed and interpreted by the assistance of the whole work team. All authors read and approved the final manuscript.
